# ﻿Gallophilous theory of cyclical parthenogenesis in aphids (Homoptera, Aphidinea)

**DOI:** 10.3897/compcytogen.18.136095

**Published:** 2024-12-17

**Authors:** Ilya A. Gavrilov-Zimin

**Affiliations:** 1 Zoological Institute of the Russian Academy of Sciences, Universitetskaya Emb. 1, Saint Petersburg, 199034, Russia Zoological Institute of the Russian Academy of Sciences Saint Petersburg Russia

**Keywords:** Adelgidae, Eriosomatidae, evolution, galls, oviparity, Pemphigidae, Phylloxeridae, unisexual (virgin) reproduction, viviparity

## Abstract

The paper elaborates theoretical basis of the origin of aphid cyclical parthenogenesis in view of the original life of these insects in strobiloid galls on *Picea* spp. The period of gall opening is greatly extended in time, which prevents normal panmixia and creates a selective advantage for parthenogenetic reproduction. Migration of aphids to secondary host plants, on which closed galls never form, parthenogenetic reproduction on these plants, and the subsequent simultaneous return of “remigrants” to the main host plant make it possible to synchronize the development of the bisexual generation and achieve mass panmixia at the end of the life cycle only; it coincides with the end of summer growth shoots or the autumn end of the vegetation period as a whole. The evolutionary transition of aphids from conifers to angiosperms in the Cretaceous period in parallel meant the possibility of development in more spacious galls accommodating several consecutive parthenogenetic generations, the transition to viviparity and telescopic embryonization, significantly accelerating the propagation.

## ﻿Introduction

Insects of the suborder Aphidinea (aphids), among all organisms, are characterized by one of the most aberrant reproductive modes, combining both extremely rare and absolutely unique features. An extensive literature is devoted to this topic, but a reader can check the most general modern reviews (Blackman 1987; Moran 1992; Hales et al. 1997; Simon et al. 2002; Wilson et al. 2003; Wool 2005; Davis 2012; Gavrilov-Zimin et al. 2015; Yan 2020; Gavrilov-Zimin 2021) to start. The complicated reproduction system of aphids is based on cyclic parthenogenesis, which combines strict, regular alternation of one bisexual generation with one or more parthenogenetic generations with the appropriate regular seasonal change of host plants.

Such alternation is an apomorphic character of the entire suborder Aphidinea and among the approximately 5000 modern species of aphids not a single one is known that does not have parthenogenetic generations. Moreover, all species of aphids known only from parthenogenetic generations are considered to have lost their bisexual generation, usually coincident with loss of the primary host plant (Mordwilko 1901; Popova 1967; Moran 1992, etc.). Cyclic parthenogenesis of aphids is operated by a unique aphidoid cytogenetic mechanism, in which exclusively parthenogenetic females emerge from fertilized eggs, whereas males and amphigonous females, on the contrary, are produced by parthenogenesis only (Blackman 1987; Dixon 1987; Hales et al. 1997; Gavrilov-Zimin et al. 2015). In the more archaic aphid superfamily Phylloxeroidea, females of all generations always lay eggs outside of their bodies, whereas in the more advanced superfamily Aphidoidea, parthenogenetic females exhibit placental viviparity. In the latter case, the situation is complicated by paedogenesis (reproduction at immature instars) and telescopic embryonization (see more about embryonization of different groups of organisms in a special article: Gavrilov-Zimin 2024), in which the embryo developing inside mother’s body already contains embryos of the next generation. The bisexual generation of aphids always differs from parthenogenetic generations in reduced fertility (down to a single egg that fills the entire abdominal cavity of the female), usually smaller in size, underdevelopment of some organs, and in many cases also complete aphagia. All these features have been discussed many times and in great detail in the aphidological literature, and by now, the theory of the connection between cyclical seasonal migrations of aphids to different host plants with the different nutritional value of these plants in different months of the warm season of the year can be considered quite well substantiated, including experimentally (Mordwilko 1901; Popova 1967; Havill and Foottit 2007, etc.). This theory connects the origin of aphids and their main diversity with life on plants of the temperate climate of the Holarctic and is in good agreement with the extreme taxonomic paucity of aphids in the tropical zone and throughout the southern hemisphere of the planet, as well as the complete absence of the most archaic groups (Adelgidae and Phylloxeridae) in the tropical climate. Here it is appropriate to cite R. Blackman and V. Eastop (https://aphidsonworldsplants.info/Introduction/): “*Cyclical parthenogenesis is a very successful way of exploiting the short-lived growth flushes of temperate plants, and aphids are thus a very successful group in temperate climates, using seasonal clues to time the alternation of the sexual and parthenogenetic phases of their life cycles. Such life cycles cannot however be readily adapted to tropical conditions. Aphids moving from temperate zones into the tropics simply lose the sexual phase of the life cycle, and in doing so they lose the potential to evolve and diversify that is dependent on the recombination of genes. The tropics may also have acted in this way as a barrier to aphid colonization of southern temperate regions, which also have very small indigenous aphid faunas*”. However, the question of the origin of the aphid cyclic parthenogenesis itself and their paradoxical reproductive system still remains open. I also cannot agree with the above quote about “very successful way” of reproduction through cyclic parthenogenesis (and parthenogenesis in general) in the conditions of a short growing season in a temperate climate, because parthenogenesis, in itself, does not lead to an acceleration of individual development and/or propagation. Such acceleration in any climate can be achieved, for example, due to the loss of some stages of ontogenesis or due to telescopic embryonization, but archaic groups of aphids (adelgids and phylloxeras) do not have these; the last feature appears only in the advanced superfamily Aphidoidea. Among the huge group of Arthroidignatha (= Hemiptera s.s.) insects, which unites about 120,000 species, not a single example of cyclic parthenogenesis is known outside of Aphidinea.

Menwhile, among Heteroptera (about 50 000 species), in Cicadinea (about 50 000 species) and Psyllinea (about 3,500 species), examples of usual thelytokous parthenogenesis occur as rare exceptions (Kuznetsova et al. 2021). In Aleyrodinea (about 1500 species), arrhenotokous parthenogenesis is known, combined with the usual regular panmixia. In Coccinea (more than 8,000 species), the sister group to aphids, parthenogenesis (of the thelytoky, arrhenotoky or deuterotoky type), with rare exceptions, is facultative and combined with irregular bisexual reproduction (Gavrilov-Zimin 2018). At the same time, true bugs, cicads, psyllids and scale insects, unlike aphids, are characterized by significant taxonomic diversity in different climatic zones of both hemispheres of the planet, including in the temperate climate of the Palaearctic, where among these groups there are species with both one and several generations per year. For example, the scale insect *Diaspidiotusperniciosus* (Comstock, 1881) (Homoptera: Diaspididae) in southern Europe has up to 5 generations per year with obligate bisexual reproduction (Danzig 1993: 192).

In general, for insects and other animals, constant bisexual reproduction is the main, absolutely dominant method of reproduction, the selective advantage of which is undoubtedly due to increased heterozygosity and a corresponding increase in the range of variability in populations. The theoretical basis for the selective advantage of bisexual reproduction has been developed in detail in numerous publications on population genetics and evolutionary theory (see, for example, Felsenstein 1974; Smith 1978; Otto 2009).

The names of higher taxa of aphids and other related insects are given according to the nomenclature-taxonomic system from Gavrilov-Zimin et al. (2015, 2021).

## ﻿Origin of cyclic parthenogenesis in Adelgidae

Since all modern species of aphids possess parthenogenetic generations in their life cycle, and it is not possible to reliably judge the nature of reproduction of extinct species, the time of transition from obligate bisexuality to heterogony can only be determined very approximately. Heie (1987: 384–385) suggested that such a transition could have occurred at the beginning of the Cretaceous. This hypothesis was based solely on the fact that among the remains of the extinct Cretaceous superfamily Canadaphidoidea (sister to modern superfamilies of aphids), individual body parts of one male are known, which supposedly had a copulatory organ: “*One among five known specimens carries a ventral process which hardly can be anything else than a male’s copulatory organ. Males are rare today, and no other male has ever been found among fossils. This may mean that parthenogenetic reproduction did not occur in Canadaphidoidea, but arose in its sister group after the separation had taken place*”. Since copulatory organs are also present in males of all modern families of aphids, it is rather difficult to understand Heie’s idea. Other authors (for example, Moran 1992) preferred vaguer statements that parthenogenesis arose very early in the evolution of aphids.

**Figure 1.**
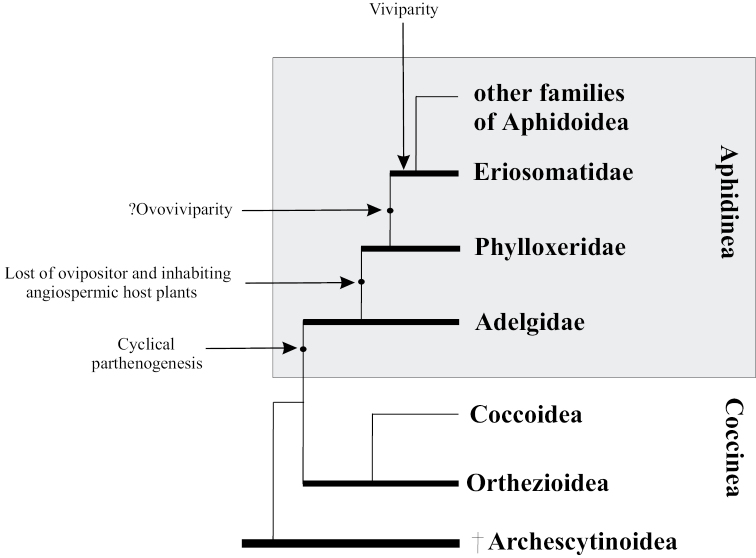
Hypothetical scheme of the evolution of reproductive peculiarities in aphids. The bold lines designate presumable paraphyletic taxa.

The first remnants of aphids are known from the Triassic period (Heie 1987) or even from the Permian period (Shcherbakov 2007). At that time, angiosperms did not yet exist. The latter became a noticeable component of ecosystems only in the Cretaceous period, although their first representatives could have appeared somewhat earlier (Han et al. 2023). Consequently, at the initial stage of their evolution, aphids were associated with gymnosperms and, possibly, with some higher spore plants. This is in perfect agreement with the fact that among living aphids, the most archaic in their reproductive biology are representatives of the family Adelgidae, obligately connected with gymnosperms. It is the adelgids that, of all aphids, have a normally developed ovipositor and are characterized by obligate oviparity in all generations of the life cycle. Oviparous aphids also include the sister family Phylloxeridae, but in them the ovipositor is lost (or represented by a vestigium) and there is not a single example of connections with gymnosperms. All other aphids (superfamily Aphidoidea) exhibit viviparity in parthenogenetic generations, complete loss of the ovipositor, and feed primarily on flowering plants (Fig. 1); their few connections with gymnosperms are clearly apomorphic (see below). Thus, it is logical to consider adelgids as a group that has preserved the original lifestyle and mode of reproduction for all aphids. Various morphological apomorphic characters of modern adelgids, of course, do not contradict the preservation of the archaic reproductive system, since the evolution of any taxon is mosaic.

**Figure 2.**
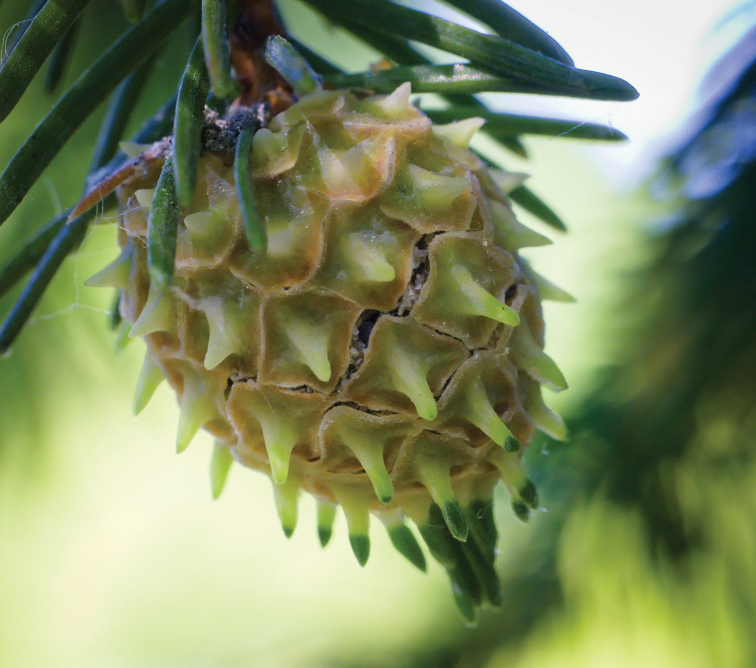
Mature gall of *Adelges* sp., Poland. Photo & copyrights: https://www.flickr.com/photos/hedera_baltica/52949379151/.

The life of aphids on bark or needles is not fundamentally different from the life of other related groups of insects living on gymnosperms, but do not have cyclic parthenogenesis (for example, coccids, true bugs, cycads). A unique feature that distinguishes adelgids from all these insects is the ability to induce the formation of closed galls, very similar and probably homologous to strobili (Fig. 2). Such galls are formed exclusively on spruce trees (*Picea* spp.), despite the regular seasonal migration of many adelgid species to other genera of gymnosperms. This means that the formation of the gall is determined not only by the special chemical composition of adelgid saliva, but by the specific response of the growing spruce shoot to the penetration of this saliva. It is noteworthy that living on the same plants (and even on the same spruce branches) monophagous soft scales *Physokermes* spp. (family Coccidae) do not cause the formation of any galls. The difference in feeding behavior between these soft scale insects and adelgids is that the former never live on annual growing shoots, while the latter, on the contrary, settle on the bud and provoke its total or partial transformation of a growing shoot into an open and then a closed gall. In this case, the gall is not of an arbitrary shape, but always resembles a spore-bearing shoot (strobilus), i.e. the transformation occurs within the usual range of morphological variation of the plant. In this regard, the galls of adelgids, unlike the galls of other aphids (and most other gall-forming animals), should not be considered as a kind of “neoplasm”, a plant “tumor”. Both the strobilus and the adelgid gall are in fact a shortened shoot with thickened needles transformed into covering scales.

**Figure 3.**
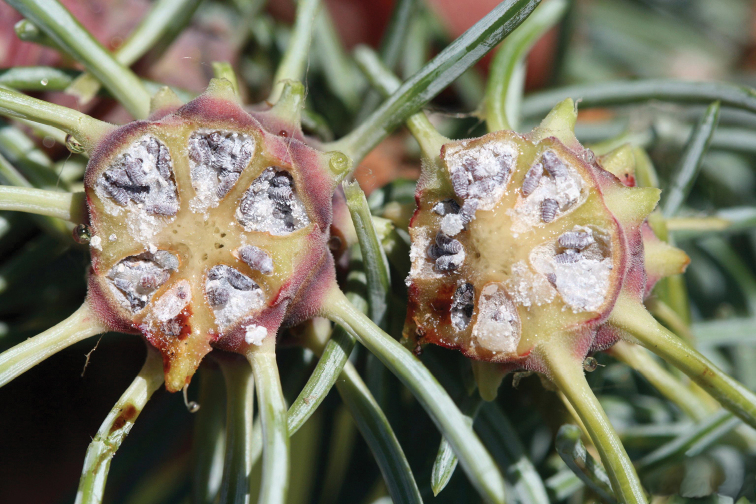
Transverse section of gall of *Adelgescooleyi* (Gillette, 1907) with nymphs inside, USA. Photo & copyrights: Whitney Cranshaw, https://www.insectimages.org/browse/detail.cfm?imgnum=5422255.

It is logical to speculate that in the early stages of aphid evolution, when the first adelgids or their ancestors began to live on the ancestors of modern spruce trees, the formation of galls occurred in the same way as now. Reliable paleontological finds attributed to the modern genus *Picea* Dietrich, 1824 have been known since close to the beginning of the Cretaceous period, 136 million years ago (Klymiuk and Stockey 2012). It is clear that the appearance of modern spruce taxa was preceded by a long evolution of their ancestral forms. Probably, even then, the spring generation of aphids was locked in the closed cavities of the galls (Fig. 3) for a period that was not and could not be strictly fixed. Spruce galls are opened (due to the drying of their “leaves”), not when the aphids sitting inside need it, but in accordance with the physiological characteristics of a particular plant and a particular tree branch. It is clear that even spruce trees of the same species growing in the same area are differently lit and shaded, are able to obtain moisture and nutrients from the soil to varying degrees, have different ages, heights, crown spans, etc. Also, different parts of the crown of the same tree are illuminated and supplied with moisture to varying degrees. As a result, the opening of adelgid galls, even those growing in the same locality, lasts for months. For example, in the vicinity of St. Petersburg (North-West Russia) this lasts from mid-June to the late August (Cholodkowski 1915; Popova 1967), and in Japan (Hokkaido) — from mid-June to early September (Tabuchi et al. 2009). It is also a well-known fact that adelgid galls “*ripe in the crown of trees (spruces) not simultaneously, but as the lower and then the upper branches mature*” (Popova 1967: 184). In some cases, according to my observations, even the opening of different cavities of the same adelgid gall does not occur simultaneously, but individual cavities of dried galls remain closed, or the resulting hole is not wide enough, and then the nymphs inside die. There are also well known examples of time-extended opening of galls in other groups of aphids. Thus, in *Phylloxeradevastatrix* Pergande, 1904 (Phylloxeridae), the galls on the shoots and petioles of hickory (*Carya* spp.) leaves open from the first half of May to mid-June (Baker 1935), in *Colophacompressa* (Koch, 1856) (Eriosomatidae) the females fly out from the galls (even on the same branches of one elm-tree) throughout July (Gavrilov-Zimin, personal observations in Leningrad Prov. of Russia), and in *Pemphigusspyrothecae* Passerini, 1860 (Eriosomatidae) the galls open from late August to early November and in some cases remain completely closed, dooming their entire population to death (Popova 1967: 108; Gavrilov-Zimin, own observations). All of these examples, as well as many others discussed below, clearly demonstrate the inconsistency of the hypothesis that aphids allegedly secrete certain chemical substances that stimulate the gall to open at the right time for the aphids (Tabuchi et al. 2009: 459): “…*opening of adelgid galls is induced by stimuli of larvae in the galls (Rohfritsch 1992), suggesting that the longer larval period is not because the larvae are waiting for the galls to open*” Moreover, the size and the speed of maturation of the adelgid gall also depends on how many fundatrices participated in its formation and how many larvae of the daughter generation (gallicolae) inhabit it (Ozaki 2000).

In the splitting approach to the taxonomy of adelgids, it is often believed galls that formed in different parts of the crown and open at different times are caused by different “cryptic” species from 7 different genera of adelgids (Cholodkowski 1915; Shaposhnikov 1964, etc.). Fortunately, in the latest taxonomic catalog of adelgids (Favret et al. 2015), the number of genera is reduced to two: *Adelges* Vallot, 1836 and *Pineus* Shimer, 1869, but the number of nominal species still remains quite large (about seventy).

It is extremely difficult to understand how most of these “species” differ from each other, since the existing identification keys (for example, Cholodkowski 1915; Shaposhnikov 1964; Mamontova 1991, etc.) are not built on clear morphological characteristics, but on vague, overlapping descriptions of lifestyle and association with a specific part of the host plant. That is, in such reasoning, galls located on different branches of the same tree open at different times because they contain aphids of different “cryptic species,” and these “species” are “independent” because their galls open at different times... No one has carried out any crossing experiments to objectively confirm the independence of these “species,” although recently (Havill et al. 2020) an attempt was made to reconstruct the pattern of hybridization between several nominal species of adelgids using indirect methods of molecular genetics. Most of the nominal “species” of adelgids have been described based on parthenogenetic lines, which, according to the authors of these species, do not engage in the sexual process (see more below). In any case there is no doubt that initially different nominal species of *Adelges* and *Pineus* were represented with a single ancestral species, because the monophyly of adelgids is doubtless and is not denied by anyone. There is also no doubt that this ancestral species initially reproduced in a normal, regular bisexual mode, like most other insects. The fundatrices then began feeding and ovipositiing on the growing spruce shoots (or spruce ancestors). The shoot grew, gradually isolating the hatched larvae inside the galls from the external environment. The larvae molted several times and grew, filling with their bodies the entire cramped space of the gall cavities (Fig. 3). The subsequent extremely unsynchronous opening of galls on different branches and on different spruce plants inevitably led to the fact that the simultaneous mass emergence of male and female nymphs from different galls was impossible. A significant or even the majority of female and male nymphs in the population always found themselves locked in the cavities of “immature” galls, while a smaller part emerged from the galls that had already opened on a particular day or several days. These circumstances led, at least, to the following consequences. 1) The small number of males and females emerging from the galls on any day or several days could not ensure effective panmixia in view of the small size of the aphids and their relatively weak abilities for independent purposeful flight. 2) Sexually mature nymphs of females, located in galls that had not yet opened, found themselves physically isolated from males that had already emerged from other galls. 3) As the appearance of adult winged females spread over time, they scattered to various suitable food plants (spruce, larches, fir, pine, etc.) and most of the females remained unfertilized. In such situation, which regularly repeated every warm season for millions of years, females capable of parthenogenetic reproduction received a selective advantage. Parthenogenesis allowed each female to not synchronize the development of her reproductive system and subsequent reproduction with the development of other individuals of the population. After early or late emergence from the gall (or even directly inside the gall, as in the modern species *Pineussimilis* (Gillett, 1907)), adelgids laid parthenogenetic eggs. These eggs hatched into larvae that either managed to feed on their own and go through one or two molts before the end of the warm season, or without feeding they crawled into cracks in the bark and overwintered there. With the onset of the next warm period of the year, larvae of different stages began feeding, moulted, and asynchronously turned into adult females and males. In this case, it again turned out to be impossible to achieve effective panmixia, and parthenogenetic females that laid eggs without fertilization again received a selective advantage in the second generation.

The fate of the third generation of adelgids that settled on the main host plant and on secondary plants turned out to be different. On spruce trees, females of the third generation began feeding on young shoots (as the most favorable place for feeding) and their progeny again found itself locked inside the galls. The change of generations on spruce trees, thus, turned out to be cyclic and exclude bisexual reproduction entirely.

On secondary host plants, sap sucking did not lead to the formation of galls and, accordingly, there were no physical obstacles to the free mating of males and females. However, due to the asynchrony of the two previous generations, the problem remained of re-synchronizing the appearance of males and females in mass numbers and their meeting in the same place, which is necessary for effective cross-fertilization. The only opportunity for such secondary synchronization was the time of approximately the same end of growth of shoots of coniferous trees by the middle of the warm season. In modern conditions, depending on the specific region and the conditions of a particular year, shoot growth ends by the end of June-beginning of July (Popova 1967). By this time, many desynchronized parthenogenetic lines of adelgids are already developing on secondary food plants. The simultaneous sharp deterioration in nutrition leads to the fact that winged “sexupares” begin to appear en masse in these lines. It is this fourth or fifth generation that moves from thelytoky to deuterotoky, that is, it forms eggs in itself, both female and male. The sexupares fly to the main host plant (spruce), where they lay eggs on the underside of the needles. From these eggs, a bisexual generation develops relatively synchronously and, finally, the possibility of mass cross-fertilization with all its genetic advantages is achieved. Under these conditions, it is important that sexuparae return from various secondary host plants to spruce, since in this way a high concentration of males and females is achieved in the same place. Due to the fact that the development of the bisexual generation always occurs in the second half of the year, when the conditions for feeding aphids on woody plants become unfavorable, the small size of the sexual individuals and their reduced fertility (often only one egg per female) are understandable, in comparison with parthenogenetic generations of the first half of the year. The loss of wings in the sexual generation, as well as in the parthenogenetic generations developing in the first half of the year on secondary plants, also becomes clear. To achieve synchronicity and mass cross-fertilization, it is important that both generations remain in their places — the first until the process of fertilization, and the second until the synchronous end of shoot growth.

**Figure 4.**
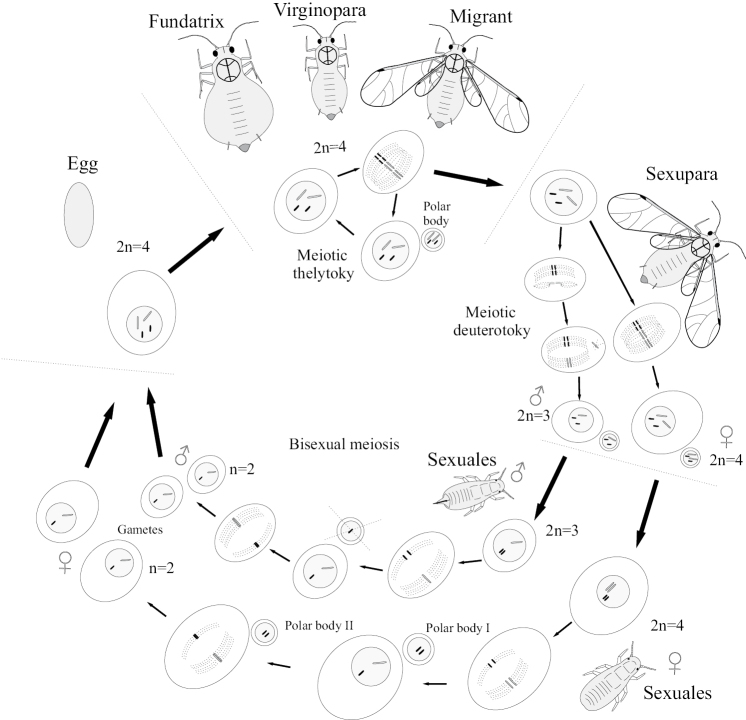
Generalized scheme of cyclical parthenogenesis in holocyclic aphids with diploid chromosome number 4 (after Gavrilov-Zimin 2021).

Thus, the occurrence of a complicated cyclical change of parthenogenetic and bisexual generations in aphids can be explained by the same basic biological reasons that appeared at the initial stage of the evolution of oogamous multicellular organisms and continue to operate up to now; namely, the problems of mass synchronous production of gametes and their concentration in the same place of space (Gavrilov-Zimin 2023). In aphids, parthenogenetic generations absolutely dominate in the life cycle. In this case, the oocytes of the parthenogenetic female undergo only one meiotic division, with the formation of a single polar body (Fig. 4); recombination of homologous chromosomes is sometimes assumed, but has not been sufficiently studied and only a few species were ivolved in such investigations (Blackman 1987). Parthenogenetic females are capable of producing embryos with a single or double set of X chromosomes, through a special cytogenetic mechanism (“mini-meiosis”) that ensures appropriate elimination of one set of X-chromosomes in oocytes, developing into male embryos (Orlando 1974; Blackman and Hales 1986).

Aphid spermatogenesis occurs in a unique way. Instead of the usual male meiosis producing four identical gametes, aphids produce only two sperm, both with X chromosomes (Blackman 1987). Thus, in the bisexual generation of aphids, all individuals turn out to be homogametic, carrying X chromosomes, and as a result of subsequent fertilization, all offspring have paired X chromosomes and become females. For this reason, the bisexual generation is necessarily replaced by parthenogenetic generation (heterogony). Probably, such a system was formed over hundreds of millions of years of forced alternation of generations (for the reasons discussed above) and was so firmly entrenched in the aphid genome that abandoning it became impossible even after more advanced groups of aphids switched to feeding on angiosperms, including those on which closed galls are not formed (see below).

Below I shall consider the real life cycles of modern adelgid species, which seem to be rather similar with the hypothetical cycle of their ancestor.

## ﻿Life cycles and ontogenesis of extant species of Adelgidae

Amongst about 70 recent nominal species of adelgids, combining in the genera *Adelges* and *Pineus*, only 24 species are considered as holocyclic (or probably holocyclic): *Adelgescooleyi* (Gillette, 1907), *A.glandulae* (Zhang, 1980), *A.isedakii* Eichhorn, 1978, *A.karafutonis* Kono et Inouye 1938, *A.kitamiensis* (Inouye, 1963), *A.knucheli* (Schneider-Orelli et Schneider, 1954), *A.lariciatus* (Patch, 1909), *A.laricis* Vallot, 1836, *A.merkeri* (Eichhorn, 1957), *A.nordmannianae* (Eckstein, 1890), *A.pectinatae* (Cholodkovsky, 1888), *A.prelli* (Grosmann, 1935) *A.roseigallis* (Li et Tsai, 1973), *A.tardoides* (Cholodkovsky, 1911), *A.torii* (Eichhorn, 1978), *A.tsugae* Annand, 1924, *A.viridis* (Ratzeburg, 1843), *Pineusarmandicola* Zhang et al., 1992, *P.cembrae* (Cholodkovsky, 1888), *P.floccus* (Patch, 1909), *P.orientalis* (Dreyfus, 1888), *P.pinifoliae* (Fitch, 1858), *P.sichunanus* Zhang, 1980, *P.strobi* (Hartig, 1839).

The detailed information on all mentioned species can be easily found on the site of R. Blackman and V. Eastop: https://aphidsonworldsplants.info/, and also in the main monographs on Nearctic and Palaearctic species: Marchal 1913; Cholodkowski 1915; Annand 1928; Popova 1967, Steffan 1968, etc.

**Figure 5.**
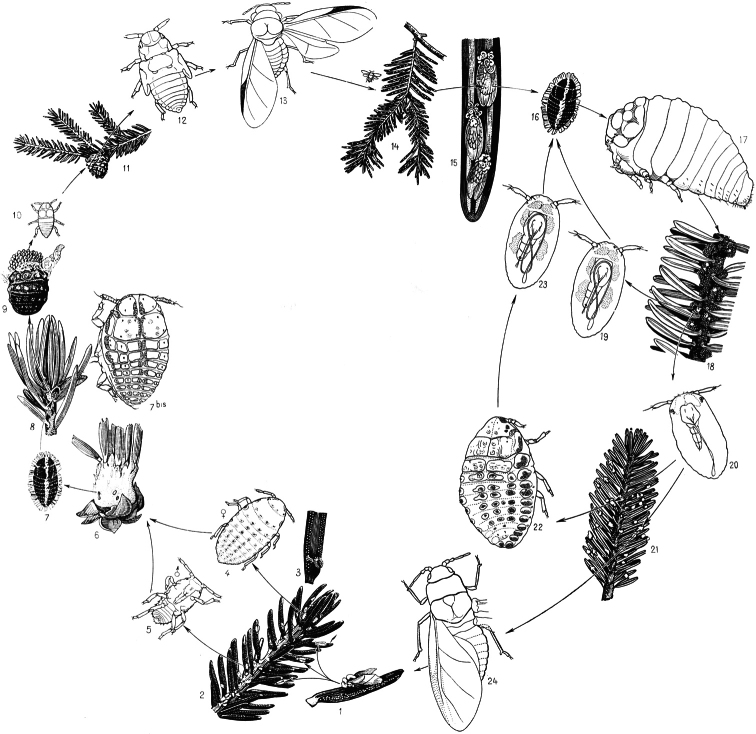
Biennial life cycle of *Adelgesnordmannianae* (Eckstein, 1890) (Aphidinea) (after Pesson 1951; Gavrilov-Zimin 2021), with changes. Stages 1–13 occur during first year on *Piceaorientalis* (Linnaeus, 1763): 1 female “sexupara”, migrated from fir (June) 2, 3 larval instars on spruce (July) 4, 5 female and male (July) 6 oviposition (July) 7 wintering larva (August-March) 7^bis^, 8 female “fundatrix” (April) 9 oviposition (April) 10, 11 larva, producing a gall on twig of spruce (Mai) 12 nymph (June) 13 migrating female (July). Stages 14–24 occur during second year on *Abiesnordmanniana* (Steven, 1838): 14, 15 females, migrating from spruce lay eggs (July) 16 wintering larva (August-April) 17, 18 parthenogenetic female and it oviposition (Mai) 19–23 new parthenogenetic generations (Mai-June) 24 alate female, migrating to spruce (June).

The larvae of these holocyclic species form closed galls on the spring shoots of various spruce species (*Picea* spp.), and during the summer the larvae emerge from the opened galls, molt for the last time and become winged migrants (Fig. 5). It is believed that among species of the genus *Adelges*, migration occurs mainly to various species of larch (*Larix* spp.), less often to various species of fir (*Abies* spp.) and very rarely (in *A.cooleyi* and *A.tsugae*) to Douglas-fir (*Pseudotsuga* spp.) and hemlock (*Tsuga* spp.). In full-cycle species of the genus *Pineus*, migration always occurs to various species of pine trees (*Pinus* spp.).

The life cycles of most of these species fully correspond to the supposed cycle (described above) of the ancestral species for adelgids and aphids in general. However, several nominal species of the genus *Pineus* (less archaic than *Adelges*) show interesting nuances and deviations. Thus, in the American nominal species *Pineusstrobi* (Hartig, 1839) (allegedly widely distributed throughout the Palaearctic), in the USA, the usual migration occurs between primary food plants (*Picea* spp.) and Weymouth pine (*Pinusstrobus* Linnaeus, 1753) (Popova 1967: 203). But there is complete confusion in the relevant literature regarding the details of the life cycle, due to the above-mentioned taxonomic chaos of aphid “species” that have no discrete differences from each other. Firstly, *Pineusstrobi* itself has no clear differences from the previously described and very widespread *P.pini* (Goeze, 1778). Secondly, the later described American “species” *P.floccus* (Patch, 1909) is no different from *Pineusstrobi* (Popova 1967: 203). All three of these supposedly “independent” species develop mainly on secondary host plants (pine trees), producing several parthenogenetic wingless generations of females per year. However, from the end of spring to mid-July, winged sexuparae appear in greater or lesser numbers and migrate to the spruce if suitable spruce species are available nearby. According to Raske and Hodson (1964), the sexuparae of *P.strobi* fly to spruce trees in late June and early July, lay eggs on the needles, but their offspring do not survive. In *P.floccus*, according to Walton (1980), generations of fundatrices, sexuparae and sexuales are probably completely absent (i.e. the life cycle is represented exclusively by parthenogenetic generations, one of which develops in galls on spruce, and the next several on pine). In the early spring of the second year, overwintered parthenogenetic females lay eggs from which both wingless parthenogenetic generations develop, remaining on the pine tree, and winged parthenogenetic females, which fly to the spruce in May, lay eggs there, and from these eggs a new generation of gall-forming larvae emerges (Walton 1980). In addition, all three of the mentioned nominal species (*Pineuspini*, *P.strobi*, and *P.floccus*) are not fundamentally different from *P.orientalis* (Dreyfus, 1888), except that the latter is characterized by a two-year life cycle typical for adelgids with a regular change of food plants (Marchal 1913; Havelka et al. 2019). Moreover, for *P.orientalis* it is known that on some species of spruce its sexual generation develops normally, while on others it dies (Popova 1967: 200). Thus, it would be logical to consider all four nominal species as a single widespread species under the oldest name *Pineuspini* s.l., and the indicated deviations in reproductive behavior to consider as a manifestation of intraspecific variability in different climates and on different food plants. A notable feature of this variable species can be considered the very early (starting from the end of May) appearance of winged females on the secondary host plant (pine trees), when the nutritional value of the growing shoots of coniferous trees is still high. This peculiarity may be due to the fact that pines often grow in arid habitats and at least in some southern parts of the extensive range of *Pineuspini* s.l. the growth of shoots of the host plant stops by the end of spring. Similar examples of the very early appearance of winged sexuparae are known for *Adelgesviridis* in the vicinity of St. Petersburg (Russia) on larches under conditions of abnormally warm spring (Popova 1967: 188).

The remaining “species” (about 50–55) from the genera *Adelges* and *Pineus* were described according to parthenogenetic generations, living on secondary host plants, less often on spruce trees (in galls or cracks in the bark). It is known about many of these “species” that in the summer winged sexuparae appear among the wingless parthenogenetic females, but their further fate has not been traced. In other cases, a detailed examination of the morphology and lifestyle makes it easy to guess from which full-cycle species the corresponding parthenogenetic lineages originate. Thus, unholocyclic *A.aenigmaticus* Annand, 1928, *A.diversis* Annand, 1928, *A.geniculatus* (Ratzeburg, 1844), *A.japonicus* (Monzen, 1929), *A.karamatsu* Inouye, 1945, *A.lapponicus* (Cholodkovsky, 1889), *A.oregonensis* Annand, 1928, and *A.tardus* (Dreyfus, 1888), as well as holocyclic *A.isedakii* Eichhorn, 1978, *A.lariciatus* (Patch, 1909), and *A.tardoides* (Cholodkovsky, 1911), in fact are the variations of *Adelgeslaricis* Vallot, 1836 – see more detail comments on R. Blackman’s and V. Istop’s site: (https://aphidsonworldsplants.info/d_APHIDS_A/#Adelges).

An interesting feature is known in the gall-inhabiting generation of *Pineussimilis* (Gillett, 1907), which supposedly develops only on spruce trees, without migrating to secondary host plants. Two variants of larvae feed in the galls. Usual larvae give rise to winged migrants that fly to the branches of the same spruce or neighboring spruce trees. Other larvae moult directly inside the gall onto wingless females, which lay eggs there (Cumming 1962). This is so far the only known example of reproduction inside galls among adelgids, although similar examples, as will be shown below, are rather often in more “advanced” groups of aphids.

## ﻿Evolution of cyclic parthenogenesis in Phylloxeridae

**Figure 6.**
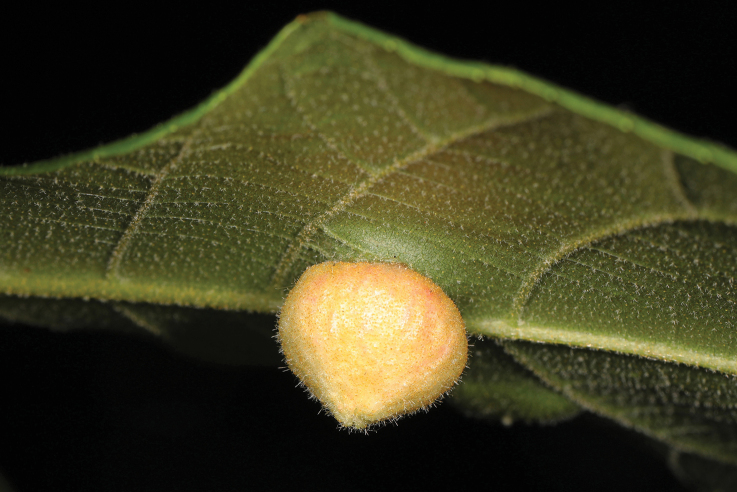
Gall of *Phylloxera* sp. on leaf of hickory, USA. Photo & copyrights: Judy Gallagher, https://www.flickr.com/photos/52450054@N04/50955746943/.

**Figure 7.**
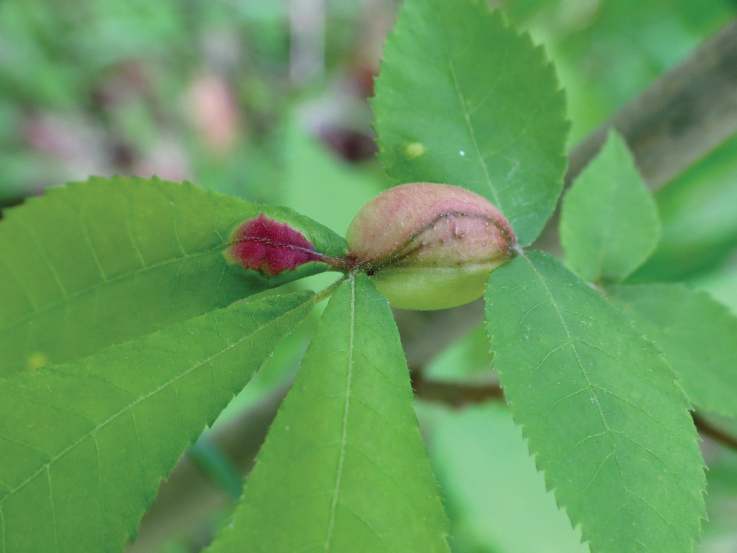
Gall of *Phylloxera* sp. on leaf of hickory, USA. Photo & copyrights: Katja Schulz, https://www.flickr.com/photos/treegrow/48516072207/.

The second group of aphids characterized by obligate oviposition in all generations is the family Phylloxeridae — phylloxeras. The cyclic parthenogenesis of these aphids, combined with the loss of the ovipositor and the complete absence of connections with gymnosperms, allows to say that phylloxeras are not just a sister group to adelgids (as is often indicated in aphidological literature, e.g. Heie 1987), but originated from a certain ancient species of adelgids after the appearance and widespread distribution of angiosperms in the Holarctic, that is, not earlier than the Cretaceous period (Fig. 1). All modern phylloxera species (about 70) are associated exclusively with woody plants, mainly beech trees (Fagales) and willows (Salicaceae). Most phylloxeras live on Nearctic flora, where they feed on various species of hickory (*Carya* spp.). On the leaf blades, petioles and twigs of these plants, phylloxera provoke galls of very diverse structure (Figs 6, 7), including closed ones (Pergande 1904; Stoetzel 1985a). In at least some of these species, the complete life cycle includes regular migration of winged females emerging from galls to secondary host plants — oaks (*Quercus* spp.) and chestnuts (*Castanea* spp.), where galls are not formed (Stoetzel 1985a). In other phylloxera species living on hickory, the development is unholocyclic or the cycle is unknown. The grape phylloxera, *Daktulosphairavitifoliae* (Fitch, 1855), which is widespread throughout the world in grape-growing areas, is also of North American origin and, moreover, is believed to descend from an ancestral species that once lived on hickory (Popova 1967: 229). On American grape varieties, this phylloxera develops as holocyclic with a regular migration from the leaves, where it lives in open galls, to the roots and back. Open galls on the edges of leaves of *Nyssasylvatica* Marschall, 1785 (Cornaceae) in the USA are also formed by *Phylloxerinanyssae* (Pergande, 1904). The species lives without host change; the bisexual generation develops in cracks in the bark. The host connection of *N.sylvatica* also appears to be of a secondary origin, since all species of the genus *Phylloxerina* Börner, 1908 are characterized by exclusively wingless generations, unholocyclic development and association mainly with willows (*Salix* spp.) and poplars (*Populus* spp.).

In the Palearctic fauna, the diversity of phylloxeras is in all respects significantly lower than in the Nearctic. Firstly, among the Palaearctic species, not a single one is known to migrate to unrelated host plants. Only for *Phylloxeraquercus* Boyer de Fonscolombe, 1834 in the Mediterranean is migration between different (evergreen and deciduous) oak species known. Secondly, with the exception of *Olegiaulmifoliae* (Aoki, 1973), which lives in closed galls on elm leaves, there are no gall-forming phylloxeras in the Palearctic. Thirdly, the range of food plants of Palaearctic phylloxeras is limited mainly to oaks, willows and poplars. One species is also known from elm, pear and chestnut. All three of these species are represented exclusively by wingless generations, as are the species living on willows and poplars. Among Palearctic species that feed on oaks, winged females are known only for a few species from the type genus *Phylloxera* Boyer de Fonscolombe, 1834 (Popova 1967: 227).

Thus, analysis of food connections and life cycles allows to conclude that the origin of phylloxeras as a taxonomic group was connected with hickories in North America. Probably, the evolutionary transition from gymnosperms, on which the adelgid ancestor of phylloxeras lived, to hickory was due to the fact that, of the Nearctic angiosperms common in the Cretaceous period, only hickory formed galls and this allowed the first phylloxeras to develop in the usual cycle of alternation of gall and freeliving generations. It is believed that *Carya* spp. appeared in North America in the second half of the Cretaceous period, and related extinct plant genera even earlier (Zhang et al. 2013). In addition to preserving the usual reproductive cycle, the life of phylloxeras in closed galls at the initial stages of their evolution apparently provided effective protection from predators and unspecialized parasites and made it possible to save from them several most proliferous generations of aphids, developing in the spring and in the first half of summer. As is well known, Adelgoidea does not have specialized hymenopteran parasites (Mamontova 2008: 109), just as almost all primitive archaeococcids (Orthezioidea) do not have such parasites, with the exception of only some species from their most “advanced” group Iceryini (Gavrilov-Zimin and Danzig 2012).

The proposed transition from living in closed galls on spruce trees to living in closed galls on hickory leaves, petioles or shoots would inevitably lead to changes in the reproductive biology of the first phylloxera. 1) Unlike the cramped internal cavities of spruce galls, hickory galls, as they grow, form a large space that far exceeds the body volume of an adult aphid. This circumstance allowed the first generation of the inhabitants of the galls not to wait for their opening, but to lay eggs just inside the gall, with the subsequent development of the second and even third generations there. In some modern species, the productivity of gall inhabitants turns out to be extremely high: in *Phylloxeradevastatrix* Pergande, 1904 — from 300 to 1300 individuals per gall, depending on its size (Baker 1935), in *Olegiaulmifoliae* — up to 1500 individuals in one gall (Shaposhnikov, 1979). 2) Laying eggs inside the gall does not require the presence of an ovipositor, since there is no need to hide the egg in any additional shelter, and thus it turns out to be possible to explain the loss of this organ in phylloxeras. 3) The development of several generations, including bisexual, inside the gall, do migration to secondary host plants unnecessary, since the life cycle with heterogony in this case is finished on the same plant. Probably for the same reason, phylloxeras does not have a two-year life cycles.

**Figure 8.**
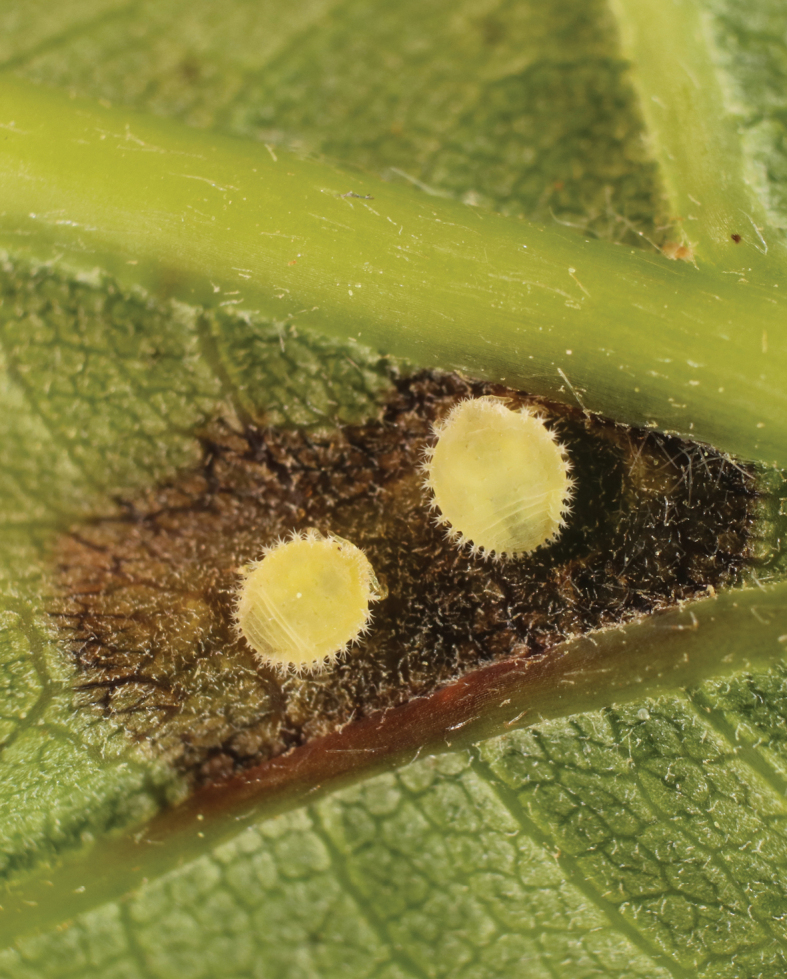
Larvae of fundatcises of *Acanthochermesquercus* Kollar, 1848 on oak leaf, Abkhazia. Photo of A.S. Kurochkin.

On the other hand, all these changes do not cancel other circumstances that played an important role in the evolution of adelgids and remained factors in the evolution of more advanced groups of aphids. These circumstances are the impossibility of synchronization and mass cross-fertilization during development in closed galls and a sharp decrease in the nutritional value of tree shoots by the middle of the warm period of the year. The action of these factors, combined with the possibility of the development of several gall generations on hickory, led to a significantly greater diversity of phylloxeras life cycles and host connections compared to adelgids. Some phylloxera species have maintained regular migrations from hickories to secondary host plants (oaks, chestnuts, and possibly some others). Other phylloxeras have switched to permanent holocyclic development on hickory. The third groups of species began to develop exclusively on secondary host plants, forming certain leaf deformations on them or forming open galls that do not interfere with the free synchronous emergence of sexuparae and/or bisexual generations. A fourth group of species, probably due to the gradual evolution of the chemistry of their saliva, began to form open galls on the hickories themselves. On the other hand, the formation of closed or open galls on hickory apparently depended not only on the evolution of phylloxeras, but also on the gradual physiological and morphological evolution of different species of these trees. It cannot be ruled out that at the very beginning of the evolution of phylloxeras, females of their common ancestor had already formed both closed and open galls, depending on what type of hickory and on what part of it (leaf blade, petiole or base of a young shoot) they started to feed. In any case, the obvious multidirectionality of the reproductive evolution of phylloxeras and the sharp expansion of their host connections in comparison with adelgids allows us to speak of their significant similarity in these parameters with Aphidoidea aphids. At the same time, neither phylloxeras nor Aphidoidea can return to constant bisexual reproduction, which was in the ancestors of aphids, due to the developed specific features of gametogenesis and the exclusively parthenogenetic method of formation of the bisexual generation (see above). Their life cycle must include at least one parthenogenetic generation, alternating with a bisexual one. Such a reproductive “minimum” is actually achieved in some modern species, for example, in the European oak phylloxera *Acanthochermesquercus* Kollar, 1848 (Fig. 8), in the cycle of which only parthenogenetic wingless fundatrices and wingless non-feeding individuals of the second, bisexual generation remain. The development of these two generations takes place in April-May, and the rest of the year the species is represented by resting eggs preserved inside the bodies of dead females (Grassi 1912: 69–70).

It is also necessary to note two interesting ontogenetic features of phylloxeras, the evolutionary significance and prevalence of which remain poorly understood. Firstly, according to the observation of M. Stoetzel (1985b), the larval instars of the bisexual generation of phylloxeras are pupal-like (“*pupiform larvae*”) due to their immobility. Such “pupae” appear after shedding the egg shell, molt four times and turn into adult mobile females and males. Secondly, for many phylloxera species, the laying of eggs of different sizes by the phyllocarcas is noted — large female and small male. Unfortunately, at present it is impossible to say with certainty whether these features are inherent in all phylloxeras and only them, or whether they are also found in some more advanced aphids (Aphidoidea).

## ﻿Further evolution of cyclical parthenogenesis and the origin of viviparity in Aphidoidea

All modern species of aphidoid aphids are characterized by the loss of the ovipositor. Taking into account this fact, reproductive characteristics and the nature of host connections, it is logical to believe that aphidoid aphids originated in the Cretaceous from a certain ancient species of phylloxeras. Otherwise, we would have to admit that the complex of characters (cyclical parthenogenesis, a unique cytogenetic system, loss of the ovipositor, the transition from gymnosperms to angiosperms) arose independently several times in the evolution of aphids; the first time in the adelgid-phylloxera branch for the reasons discussed above, and at least twice in aphidoid aphids for some other unknown reasons. Such an extraordinary combination of a number of evolutionary coincidences seems absolutely incredible. All the few connections between aphidoid aphids and gymnosperms are clearly of a secondary nature. Such connections are found in a number of genera of lachnids (Lachnidae), in representatives of the genus *Neophyllaphis* Takahashi, 1920 (Drepanosiphidae) and in some genera of eriosomatids (Eriosomatidae). These examples require somewhat more detailed consideration.

Lyachnids of the subfamily Cinarinae, widespread in the Holarctic and associated with various species of pines (*Pinus* spp.), spruce (*Picea* spp.), fir (*Abies* spp.), larch (*Larix* spp.), and cypress (Cupressaceae), are considered either by different aphidologists as one of the youngest, most advanced groups of aphidoid aphids, or, conversely, as one of the most archaic (see review of competing opinions in Mamontova 2008). These contradictions are due to the fact that the morpho-anatomical characteristics of lachnids give a mosaic picture of plesiomorphy vs. apomorphy. However, none of the aphidologists consider lachnids to be more archaic and ancient in comparison with adelgids and phylloxeras. Moreover, within the family Lachnidae itself, aphids associated with angiosperm trees (type subfamily Lachninae) appear to be more primitive than cynarines based on their morphological characters (Mamontova 2008: 104–164). In any case, all lachnids are characterized by telescopic embryonization based on placental viviparity of parthenogenetic generations. Not a single example of oviparous parthenogenetic generations is known among lachnids (as well as all aphidoid aphids) and, accordingly, it is impossible to discern a direct evolutionary connection between them and any oviparous species ancestral to all aphids.

Aphids of the genus *Neophyllaphis* of the monotypic subfamily Neophyllaphidinae are represented in the modern fauna by 18 species associated with gymnosperms of the families Podocarpaceae and Araucariaceae, mainly in the Southern Hemisphere, including in the mountainous regions of the tropical zone of the planet. All these species develop unholocyclically, but in a number of cases they demonstrate holocycly, with the appearance of winged (rarely wingless) individuals of the bisexual generation (Blackman & Eastop, https://aphidsonworldsplants.info/d_APHIDS_N/#Neophyllaphis). The very fact that these aphids, like their host plants, in their distribution are separated from the obvious center of diversity and origin of aphids, i.e. from the temperate climate zone of the Holarctic, does not in itself allow us to consider them an ancestral group in relation to other aphidoid aphids. For one of the species, *N.brimblecombei* Carver, 1971, a feeding relationship with eucalyptus (*Eucalyptusrobusta* Smith, 1792) was indicated in southern China, where the species was apparently introduced from Australia (Qiao et al. 2001); it may additionally testify the secondary nature of the connection between these aphids and gymnosperms. The parthenogenetic generations of all these aphid species are characterized by obligate viviparity, which, as in the case of lachnids, excludes a direct evolutionary connection of *Neophyllaphis* spp. with the hypothetical ancestors of aphids.

Phylogenetic reconstructions proposed by various authors for other groups of aphidoid aphids are extremely contradictory (Heie 1987; Shaposhnikov 1987; Wegierek 1992; Wojciechowski 1992; Heie and Wegierec 2009; Ortiz-Rivas and Martinez-Torres 2010; etc.) and do not allow to make unambiguous judgments about aphid evolutionary history. Based on morpho-anatomical characters, Eriosomatidae (= Pemphigidae) are usually considered as one of the archaic families of aphidoid aphids (Shaposhnikov 1987; Heie and Wegierec 2009). However, when constructing evolutionary reconstructions, none of the aphidologists-phylogeneticists pay attention to the fact that only for eriosomatids, among all Aphidoidea, the birth of parthenogenetic offspring was noted in shells, which were soon discarded by the hatched larva (Mordwilko 1901: 58; Hille Ris Lambers 1950). Since eriosomatids have the same number of larval instars as other aphids, there is no reason to assume any additional “embryonic molt”. So, these shells are of maternal origin and are homologous to the egg membrane, i.e. chorion, as Mordwilko (1901: 58) directly wrote about. This peculiarity should probably be considered as plesiomorphic, indicating a transition from ovoviviparity to placental viviparity. In general, ovoviviparity is a common intermediate stage between oviparity and viviparity in the evolution of various groups of animals, including the sister group of scale-insects (Ivanova-Kazas 1995; Ostrovsky et al. 2016; Gavrilov-Zimin 2022). In addition, the development of embryos in the body of the migrating generation in the studied eriosomatids does not occur sequentially, as in other Aphidoidea, but simultaneously, so that a female that has flown to a secondary host plant lays all her offspring in a very short period of time, just as many oviparous animals do, in particular, females of oviparous and ovoviviparous scale-insects (Coccinea). This feature of pemphigids was previously noted by Hille Ris Lambers (1950). I myself verified the simultaneity of embryo development by dissecting migrating females in such species as *Colophacompressa* (Koch, 1856), *Prociphilusfraxini* (Fabricius, 1777), *P.xylostei* (De Geer, 1773), and *Pemphigusspyrothecae* Passerini, 1860. Considering that the placental viviparity of aphids, combined with pedogenesis and telescopic embryonization, could hardly have arisen suddenly, it is logical to recognize the eriosomatids as a possible “transitional form” in the evolution from the oviparous Phylloxeroidea to the viviparous Aphidoidea (Fig. 1).

**Figure 9.**
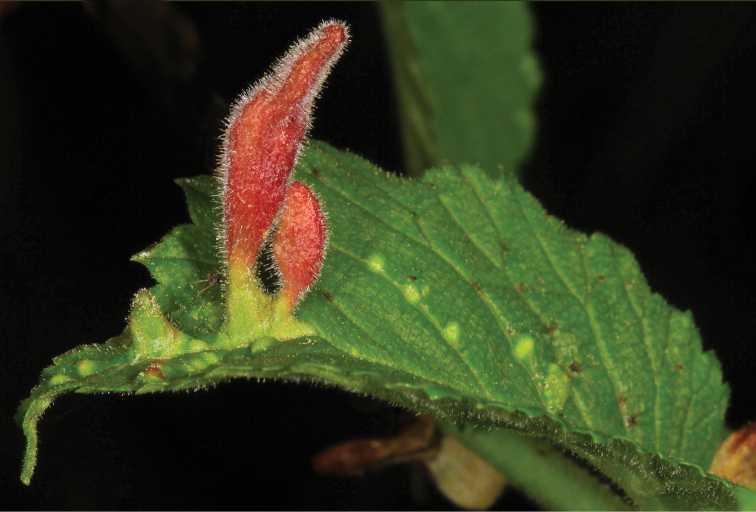
Galls of *Tetraneuraulmi* (Linnaeus, 1758) on elm leaf, USA. Photo & copyrights: Judy Gallagher, https://www.flickr.com/photos/52450054@N04/33994074962/.

The family Eriosomatidae is divided into three subfamilies: Eriosomatinae, Fordinae and Pemphiginae. Aphids of Eriosomatinae (genera *Aphidounguis* Takahashi, 1963, *Byrsocryptoides* Dzhibladze, 1960, *Colopha* Monell, 1877, *Colophina* Börner, 1931, *Eriosoma* Leach, 1818, *Gharesia* Stroyan, 1963, *Hemipodaphis* David et al., 1972, *Schouteden*, 1906, *Paracolopha* Hille Ris Lambers, 1966, *Schizoneurata* Hille Ris Lambers, 1973, *Schizoneurella* Hille Ris Lambers, 1973, *Siciunguis* Zhang et Qiao, 1999, *Tetraneura* Hartig, 1841, *Zelkovaphis* Barbagallo, 2002) mainly use as their primarily host plants various *Ulmus* spp. and *Zelkova* spp., on the leaves of which they form closed or open galls (Fig. 9). Several generations of parthenogenetic females develop in the galls. By mid-summer, winged females appear in the galls and migrate to the roots (less often above-ground parts) of various woody or herbaceous flowering plants. Several parthenogenetic generations also develop on secondary host plants, but never forming galls on these plants. In autumn, winged remigrants return to elms, where they hatch bisexual generation larvae in cracks of the bark. These larvae lack mouthparts, do not feed, molt four times, and then mate and each female lays one overwintering egg.

**Figure 10.**
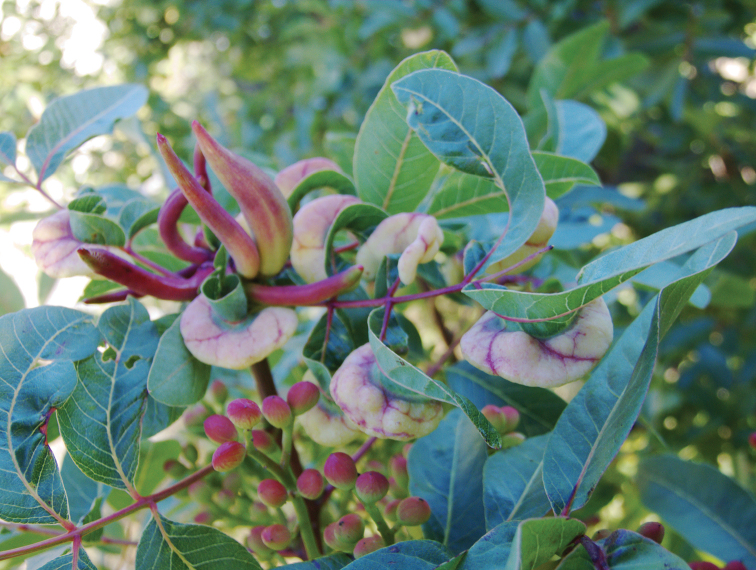
Galls of two different species of Fordinae on twigs of *Pistaciaterebinthus* Linnaeus, 1753. Photo & copyrights: Gene Selkov, https://www.flickr.com/photos/selkovjr/45002126051/.

For the subfamily Fordinae (genera *Aloephagus* Essig, 1950, *Aploneura* Passerini, 1863, *Asiphonella* Theobald, 1923, *Baizongia* Rondani, 1848, *Chaetogeoica* Remaudière et Tao, 1957, *Dimelaphis* Zhang, 1998, *Floraphis* Tsai et Tang, 1946, *Forda* von Heyden, 1837, *Geoica* Hart, 1894, *Geopemphigus* Hille Ris Lambers, 1933, *Inbaria* Barjadze et al., 2018, *Kaburagia* Takagi, 1937, *Meitanaphis* Tsai et Tang, 1946, *Melaphis* Walsh, 1867, *Nurudea* Matsumura, 1917, *Paracletus* von Heyden, 1837, *Qiao* Hébert et al., 2022, *Rectinasus* Theobald, 1914, *Schlechtendalia* Lichtenstein, 1883, *Slavum* Mordvilko, 1927, *Smynthurodes* Westwood, 1849, *Tramaforda* Manheim, 2007) the primarily host plants are *Pistacia* spp. and *Rhus* spp. Closed or open galls are formed on the leaves of these plants (Fig. 10). Life cycles are similar to those of Eriosomatinae, but the taxonomic diversity of secondary host plants is much wider and includes (in species of the American genus *Melaphis*) even mosses. In many species of Fordinae, the opening of the galls occurs only at the end of summer or even in autumn (in September-October). For this reason, the whole life cycle extends over two years. In some Fordine species, the founders first form a small “temporary” gall, and then a significant part of the females of the daughter generation leave the maternal gall and form new, more spacious galls on the same plant (Wool 2005: 87–88). Species distributed in regions where pistachios and sumacs are currently absent are represented only by parthenogenetic generations on secondary host plants. Among such populations, cases of mosaicism are sometimes encountered, when winged parthenogenetic females feeding on the roots of secondary host plants contain both thelytocous (with mouthparts) and bisexual (without mouthparts) embryos (Mordwilko 1901: 83, 214; Popova 1967: 117). Gavrilov-Zimin (2024) proposed to call this phenomenon as “mosaic embryonization.”

**Figure 11.**
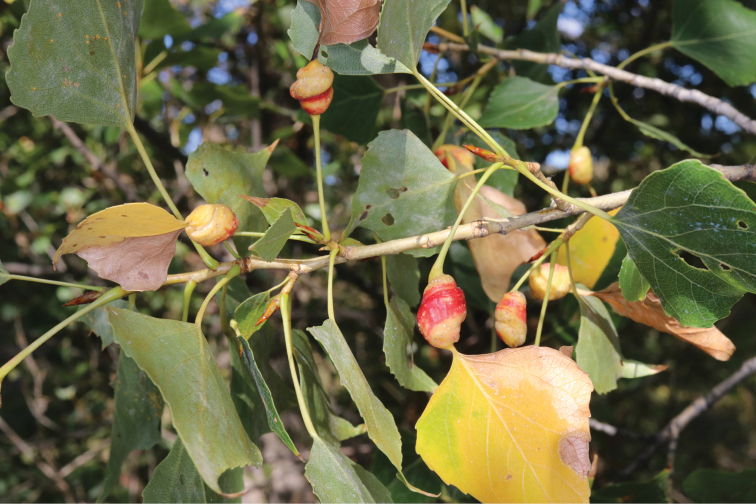
Galls of *Pemphigusspyrothecae* Passerini, 1860 on petioles of poplar, Samara Prov. of Russia. Photo of A.S. Kurochkin.

The aphids of the subfamily Pemphiginae (genera *Ceratopemphigiella* Menon et Pawar, 1958, *Ceratopemphigus* Schouteden, 1905, *Clydesmithia* Danielsson, 1989, *Cornaphis* Gillette, 1913, *Diprociphilus* Zhang et Qiao, 1999, *Epipemphigus* Hille Ris Lambers, 1966, *Formosaphis* Takahashi, 1925, *Furvaphis* Hong, 2002, *Gootiella* Tullgren, 1925, *Grylloprociphilus* Smith et Pepper, 1968, *Mimeuria* Börner, 1952, *Mordwilkoja* Del Guercio, 1909, *Neopemphigus* Mamontova et Kolomoets, 1981, *Neoprociphilus* Patch, 1912, *Pachypappa* Koch, 1856, *Pachypappella* Baker, 1920, *Patchiella* Tullgren, 1925, *Pemphigus* Hartig, 1839, *Prociphilus* Koch, 1857, *Thecabius* Koch, 1857, *Tiliphagus* Smith, 1965, *Uichancoella* Calilung, 1975) use mainly *Populus* spp. as primarily host plants, but sometimes inhabit also the other arboral angiosmerms. Spring generations feed inside closed or open galls on the leaves or petioles of poplar leaves, and winged migrants, emerging from the galls, usually fly to the roots of coniferous trees, less often to herbaceous angiosperms. The genus *Prociphilus* differs from other genera of the subfamily in an unusually wide range of primary host plants (from the families Rosaceae, Caprifoliaceae, Oleaceae, etc.), but summer migration is still carried out to the roots of coniferous trees. Some species, for example, *Pemphigusspyrothecae* Passerini, 1860, which lives in closed galls on poplars (Fig. 11), have a cycle without changing of host plants. Only a few pemphigines live in tropical climate, such as the monotypic genus *Ceratopemphigus*, whose members form closed galls on *Ligustrumrobustum* (Roxburgh, 1832) in southeast Asia; the life cycle of this species is unknown (Cock et al. 2010).

Thus, in all three subfamilies of Eriosomatidae, many species demonstrate an archaic life cycle, characteristic of adelgids, with development inside closed galls. In the subfamily Fordinae a number of species even demonstrate a two-year cycle, again characteristic of adelgids. This fact further illustrates the extreme dependence of the aphid life cycle on the specific gall formation on specific plants. It is impossible to imagine that such a prolongation of the cycle would be evolutionarily beneficial for the corresponding aphid species, in any way “controlling” the development of the gall. However, it is quite logical to explain this situation by the simple impossibility of leaving the closed galls before the end of the summer season. The appearance of placental viviparity and telescopic embryonization, which occurred for the first time probably among eriosomatids, makes it possible to significantly accelerate the change of generations, and, consequently, increase the number of descendants, regardless of the time of gall opening. Of course, this is only possible if there is sufficient internal space in the gall, which also depends on the exact host plant peculiarities.

Among other aphidoid aphids, life in closed galls is known only for a number of genera/species of Hormaphididae, assigned to the tribe Cerataphidini (Aoki and Kurosu 2010). Gall-forming cerataphidins use *Styrax* spp. trees as primary host plants, on which they form single-chambered or multichambered closed galls. Bamboo, palm and ginger plants are usually used as secondary host plants. In temperate climate, the cycle of gall-forming cerataphidins is quite similar to that of eriosomatids (Aoki and Kurosu 2010). This similarity is not surprising, given that hormaphidids are considered by some researchers to be a group closely related or even sister to eriosomatids (Heie 1987; Wojciechowski 1992). In the Asian subtropics and tropics, the cerataphidin cycle either becomes incomplete on secondary (less often primary) host plants, or migration from secondary to primary plants becomes facultative (Aoki and Kurosu 2010). The galls of *Ceratoglyphinastyracicola* (Takahashi, 1921) on *Styraxsuberifolius* Hooker et Arnott, 1837 in Taiwan reach an extraordinary longevity (up to 20 months!); the gall population can reach 100,000 individuals, approximately half of which are non-breeding individuals — “soldiers” performing a guard function (Aoki and Kurosu 2010).

In aphidoid aphids of the families Aphididae, Drepanosiphidae, Mindaridae, some species form different “pseudogalls”, which are curled leaves or needles of host plants. Such shelters do not pose problems for the free exit of migrating winged individuals of aphids, and this exit occurs as the growth of the shoots of the corresponding plants ends and their nutritional value decreases (Popova 1967).

## ﻿Conclusion

From the above consideration of the life cycles and reproductive peculiarities of aphids, it is clear that the evolution of their archaic groups: adelgids, phylloxeras, eriosomatids, and hormaphidids is fully or partially associated with life in closed galls formed on gymnosperms or angiosperms. Living in closed galls fundamentally distinguishes these aphids from other related groups of hemipteroid gall-forming insects: scale-insects, psyllids and some true bugs; among these groups, there are no examples of the formation of closed galls nor examples of cyclical parthenogenesis, although other (non-cyclical) variants of parthenogenesis are quite common (especially in scale-insects) (Gavrilov 2007; Kuznetsova et al. 2021). Closed galls, known for a few thrips species, usually crack before sexual maturation of the first gall generation (Kranz et al. 2002) and, thus, there are no problems with free exit of insects to mate with individuals from other galls. Cyclical parthenogenesis in thrips is not known, although the group as a whole is characterized by arrhenotokic parthenogenesis and haplodiploidy (Kuznetsova et al. 2021).

In this article, it is not possible or necessary to consider the remaining numerous groups of gall-forming animals, but it can be noted that among terrestrial animals, regular cyclical parthenogenesis has been proven only for some gall wasps (Hymenoptera: Cynipidae) living in closed galls (Stone et al. 2002; Csóka et al. 2005). Unlike hemipteroid insects, gall wasps, like most other gall-forming animals, are characterized by gnawing mouthparts. For this reason, their larvae and/or adults can theoretically gnaw through plant tissue and ensure their escape at any time. However, in reality, the emergence of wasps of the same species from galls in the same area is usually significantly extended over time. In a number of species this occurs only after wintering in a dead gall. Thus, the synchrony of the appearance of adult individuals is greatly disrupted. For example, in experiments with *Andricusquercuslanigera* (Ashmead, 1881) in Texas, emergence of the parthenogenetic generation from oak galls occurred from September 9 to February 24 (Hood et al. 2018). As a result, just like aphids, gall wasps exhibit a wide variety of life cycle options, which can include regular obligate alternation of parthenogenetic and bisexual generations, or be limited to only parthenogenetic generations or only bisexual, ending within one year or stretching over two years, be combined with a change of host plants or not, etc. (Stone et al. 2002).

More or less regular heterogony is also known in a number of groups of primary aquatic animals, for example, in some trematodes, rotifers and crustaceans (White 1973) and, quite obviously, arose in these groups for some other reasons that differ from those described above for aphids.

The frequent reference in the review literature (see, for example, White 1973; Gokhman and Kuznetsova 2017) to the presence of cyclical parthenogenesis in gall midges (Diptera: Cecidomyiidae) — for example, in subfamilies Porricondylinae and Lestremiinae — actually refers to the facultative appearance of bisexual generations, which does not have a regular character. The point is that representatives of some genera of gall midges reproduce primarily by paedogenesis, but a small part of their larvae can undergo complete metamorphosis and become capable of bisexual reproduction (Ivanova-Kazas 1981).

Just as often and erroneously, the life cycle of the beetle *Micromalthusdebilis* LeConte, 1878 is cited as an example of cyclical parthenogenesis. However, the reproduction of this species is carried out exclusively by parthenogenesis (Perotti et al. 2016).

Summarizing the results of the discussion, we can highlight the following main theses characterizing the evolution of the reproductive characteristics of aphids.

1. Cyclical parthenogenesis of aphids is a special variant of heterogony (alternation of parthenogenetic and bisexual generations), strictly associated with the change of seasons in temperate climates and caused by the obligate birth of thelytokous females from fertilized eggs.
2. The origin of such a life cycle can be explained by the long (millions of years) evolution of the most archaic group of recent aphids — adelgids on their main host plants (*Picea* spp. or the ancestral plant taxa), starting from the Triassic or Jurassic periods. Feeding of the spring generation of adelgids on developing spruce shoots always causes the formation of closed strobiloid-like galls, the opening of which is extremely extended in time and prevents panmixia in populations.
3. Non-synchronous opening of galls disrupts the initial synchrony of development of individuals in the population and leads to the simultaneous existence of all stages of ontogenesis during the summer period. Subsequent secondary synchronization of ontogeneses is possible only in the second half of summer on secondary or primary host plants under conditions of cessation of plant shoot growth.
4. The evolution of other archaic groups of aphids: phylloxeras, eriosomatids, and hormaphidids is also fully or partially associated with life in closed galls, but on angiosperms. Such galls, unlike galls on spruce trees, have a much larger internal cavity, which allows several parthenogenetic generations to develop inside them.
5. The loss of the ovipositor in phylloxeras (and the aphidoid aphids hypothetically descended from them) can be explained precisely by the original life in galls, where egg laying does not require special adaptations.
6. The evolutionary transition from oviparity of parthenogenetic generations to viviparity probably occurred in the ancestors of modern Eriosomatidae, as evidenced by the plesiomorphic features of the reproductive biology of the latter.
7. The appearance of placental viviparity and telescopic embryonization, which occurred probably among gall-forming eriosomatids, made it possible to significantly accelerate the change of generations, and, consequently, increase the number of descendants, regardless of the time of opening of the galls.
